# Photothermal Desorption of Toluene from Carbonaceous Substrates Using Light Flash

**DOI:** 10.3390/nano12040662

**Published:** 2022-02-16

**Authors:** Evan L. Floyd, Jonghwa Oh, Karim Sapag, Toluwanimi M. Oni, Jacob S. Shedd, Claudiu T. Lungu

**Affiliations:** 1 Department of Environmental Health Sciences, The University of Alabama at Birmingham, Birmingham, AL 35294, USA; evan-floyd@ouhsc.edu (E.L.F.); jonghwa@uab.edu (J.O.); jshedd91@uab.edu (J.S.S.); 2 Department of Occupational and Environmental Health, University of Oklahoma Health Sciences Center, Oklahoma, OK 73126, USA; toluwanimi-oni@ouhsc.edu; 3 Department of Physics, National University of San Luis, San Luis D5700HHW, Argentina; sapag@unsl.edu.ar

**Keywords:** photothermal desorption, toluene, carbonaceous substrates, thermal desorption, carbon nanotubes

## Abstract

Millions of workers are occupationally exposed to volatile organic compounds (VOCs) annually. Current exposure assessment techniques primarily utilize sorbent based preconcentrators to collect VOCs, with analysis performed using chemical or thermal desorption. Chemical desorption typically analyzes 1 µL out of a 1 mL (0.1%) extraction volume providing limited sensitivity. Thermal desorption typically analyzes 100% of the sample which provides maximal sensitivity, but does not allow repeat analysis of the sample and often has greater sensitivity than is needed. In this study we describe a novel photothermal desorption (PTD) technique to bridge the sensitivity gap between chemical desorption and thermal desorption. We used PTD to partially desorb toluene from three carbonaceous substrates; activated carbon powder (AC-p), single-walled carbon nanotube (SWNT) powder (SWNT-p) and SWNT felts (SWNT-f). Sorbents were loaded with 435 ug toluene vapour and irradiated at four light energies. Desorption ranged from <0.007% to 0.86% with a single flash depending on substrate and flash energy. PTD was significantly greater and more consistent in SWNT-f substrates compared to AC-p or SWNT-p at all irradiation energies. We attribute the better performance of SWNT-f to greater utilization of its unique nanomaterials properties: high thermal conductivity along the nanotube axis, and greater interconnection within the felt matrix compared to the powdered form.

## 1. Introduction

Every year millions of tons of volatile organic compounds (VOCs) are released into the environment by anthropogenic sources globally and domestically [1,2,3] with a portion of these emissions resulting in occupationally exposed workers. Validated exposure assessment methods [4,5,6,7] have been developed to ensure occupational exposures are below limits established by the Occupational Safety and Health Administration (OSHA), National Institute of Occupational Safety and Health (NIOSH), and American Conference of Governmental Industrial Hygienists (ACGIH), among others. Most of these methods are based on air sampling in which VOCs are preconcentrated in the field on a sorbent and brought back to a lab for analysis.

An effective sorbent captures VOCs on its surface through physical adsorption (i.e., physisorption) using weak molecular interactions. In many cases the preferred sorbent for VOC sampling is high surface area, highly microporous activated carbon (AC). AC samplers have good analyte stability, are compact, inexpensive and can be used in active or passive sampling. Active sampling generally has better sensitivity than passive sampling because a greater volume of air can be sampled but requires a calibrated pump and tubing between the pump and sampler. Passive sampling relies on chemical diffusivity to draw the contaminant inside the sampler for capture by the sorbent. Passive sampling is convenient because samplers are small, lightweight and do not interfere with worker activity to the degree of active sampling with pumps and sample lines. However, due to the lower sampling rate of passive samplers, they are not appropriate for short term, low concentration exposure assessments.

Currently, sorbent samples are prepared for analysis by chemical desorption or thermal desorption. Chemical desorption has inherent sensitivity limitations due to the small fraction of extract injected into the instrument and potential for solvent masking of early eluting compounds [8]. For example, NIOSH method 1500, 1501 [5] and OSHA method 111 [6] extract the sorbent in 1 mL carbon disulfide (CS_2_) and inject 1 µL into a gas chromatograph (GC). This represents a 1000-fold dilution of the collected sample. Similarly, NIOSH method 4000 (for passive samplers) extracts in 1.5 mL CS_2_ and injects 5 µL to a packed column, a 300-fold dilution. Since packed columns have widely been replaced by the more efficient, higher resolution capillary column many labs performing passive sampler analysis use a “modified NIOSH 1501” or “modified NIOSH 4000” method that adapts either technique to be used with passive samplers, a 1–2 µL injection, and a capillary column. Typical limits of quantification (LOQ) for commercial labs performing passive sampler analysis are 1–5 µg per sample. For full shift, occupational sampling these LOQs are sufficient, but for short duration, low concentration, passive sampling, they are insufficient. Either the instrumentation or preparation technique must be improved to reliably use passive samplers for short duration exposure assessments.

Thermal desorption is certainly an improved preparation technique that uses heat to release the adsorbed compounds from the collection sorbent. As the sample is desorbed, it is temporarily collected on a focusing trap until the entire sample is completely desorbed from the original sorbent. After which, the focusing trap is quickly heated and the sample sent to the GC column for separation and analysis. The sensitivity of thermal desorption is excellent since the entire sample can be delivered to the instrument, but in most occupational situations this is far too much sensitivity that leads to upper LOQs (limits of saturation) around 1–2 µg per sample. Splitting the sample so that only a portion (1–10%) is delivered to the instrument can be achieved by either the thermal desorption unit or the GC inlet. In the former case recapture of the split portion on another sorbent tube is possible, but is not a standard option on all thermal desorption units. In the latter case, the split portion is wasted. A sample introduction technique that bridges the sensitivity gap between chemical desorption with liquid injection (0.1%) and thermal desorption (100%) without requiring a re-capture system or wasting a large portion of the original sample would be a welcome improvement.

In this study, we explored the use of high intensity photo flash to thermally desorb toluene from three sorbents; activated carbon powder (AC-p), single-walled carbon nanotube powder (SWNT-p) and single-walled carbon nanotube felt (SWNT-f). Toluene was selected as an analyte of interest for proof-of-concept testing due to its common presence in industry, its similarity to more hazardous aromatic VOCs like benzene, and its ability to be physisorbed onto carbonaceous sorbents [9,10,11,12]. While single-walled carbon nanotubes (SWNT) were chosen as a sorbent candidate based on their high surface area [13,14,15,16,17], ability to be functionalized to alter chemical affinities [18,19,20,21], and emerging potential as environmental samplers [22,23,24,25,26]. Up to this point, use of SWNTs as VOC preconcentrators has utilized the high surface area and small pore structure inherent to SWNT, and the long aspect ratio that allows formation of felts (also known as bucky papers) [22,26,27,28,29], or the ability to grow SWNT how and where desired [23,25,30,31,32,33]. To date, desorption of these preconcentrators has been through conventional thermal means. We propose using high-intensity light that is directly absorbed by the sorbent and converted to heat to release the sorbed analyte. This approach further leverages material advantages of SWNTS; which are their high visible-light absorption property [34,35,36,37,38,39], their exceptional thermal conductivity [40,41,42,43,44], and their thermal stability across the photo flash temperature range [45].

Conventional thermal desorption heats the sampler from the outside-in. An external heating element conducts heat through the sampler body to the sorbent which heats up and desorbs the analytes. Desorption occurs relatively slowly and must be captured by a focusing trap in order to make a sharp injection into the GC for a uniform chromatographic starting point. Using high-intensity visible light reverses the heat flow. Light energy is absorbed by the sorbent and converted into heat within the sorbent matrix, causing analyte desorption. Heat now flows from the sorbent to sampler body to the environment and heat radiates directly from the sorbent surface in the infrared spectrum. Using light flash allows precise control of the total energy applied to the sorbent and the duration of energy application (micro–milliseconds); this in turn dictates the temperature rise of the sorbent and the time spent at elevated temperature. With this approach, a portion of the adsorbed analyte can be released in a sharp pulse that is ready to be delivered directly to the GC without need of a focusing trap.

Our hypothesis is twofold. (1) Irradiation with high-intensity light flash can be used to achieve rapid, reliable, partial desorption that bridges the sensitivity gap of chemical and thermal desorption techniques. (2) SWNT-f will release a significantly larger fraction of analyte than AC-p or SWNT-p when irradiated under the same conditions because SWNT-f better utilizes the long aspect ratio and thermal conductivity of carbon nanotubes through the interconnected structure of the felt. This is expected to better conduct thermal energy from the felt surface into the bulk of the sorbent and more uniformly release adsorbed analyte from the sorbent matrix.

## 2. Materials and Methods

Samples of AC-p, SWNT-p and SWNT-f were loaded with 435 ug toluene vapour (0.5 μL) and irradiated with light flash at four different energies. Desorbed toluene was quantified by a field grade photoionization detector (PID) and desorbed mass compared across flash energies and substrates. A small aluminum test chamber with internal dead volume of 16.1 mL was constructed with a borosilicate glass window, gas flow ports and an aerosol filter installed on the effluent line to capture potential fugitive carbon particulate. Dry nitrogen (N_2_) was supplied through the chamber at 300 mL min^−1^ and light flashes were applied once per minute for a total of 10 flashes at each condition. Nitrogen adsorption was used to measure surface properties of the substrates used. Toluene adsorption isotherms were obtained to directly compare sorbents’ adsorption capacity for the analyte of interest. Toluene was selected as a representative VOC because of its similarity to benzene and its use in similar studies [9,10,11,22,30,44,45,46].

### 2.1. Health and Safety Considerations

Due to potential negative health effects of SWNT, special care was taken when handling SWNT powder. All handling, transferring and weighing was conducted in a fume hood with the researcher wearing a half-mask respirator with P-100 aerosol filters, gloves and lab coat. Used substrates were recycled where possible or disposed though the university hazardous waste disposal program by incineration.

### 2.2. Sorbent Specifications

AC-p was obtained from Sigma (batch # 076K0676). SWNT-p was purchased as powder from M.K. Nano (90%+ SWNT, lot SCN0109) with the following specifications: tube diameter 1.4–2.1 nm, greater than 90% SWNT, less than 3% metal catalyst, less than 7% amorphous carbon or multi-walled carbon nanotubes. This material was used as received for SWNT-p trials and processed into felt for SWNT-f trials, as described below.

### 2.3. Substrate Construction

Felt making was based on the method described by Zheng et al. [21]. Briefly described, 20 mg SWNT-p was suspended in 150 mL toluene by bath ultrasonication for 30 min and vacuum filtered over a 37 mm silver membrane filter (0.8 µm, 225-1801, SKC). SWNT deposition area was restricted to a diameter of 26 mm due to the filtration apparatus and the felt was allowed to dry while under vacuum. Disk-shaped copper sample trays were made to contain 20 mg AC-p and SWNT-p spread over a 26 mm diameter surface, to match the surface area of the SWNT felts. Since the SWNT selected did not allow formation of self-supporting felts, SWNT-f was evaluated directly on the filtration membrane (as deposited) with the entire membrane placed on top of a copper sample tray for analysis. All substrates were desorbed at 200 °C overnight and blank verified prior to initial use. Blank verification consisted of loading a sample into the test chamber and irradiating 10 times at the maximum flash energy. Any sample that yielded a detectable response was considered not-blank. All samples were blank after overnight desorption treatment.

### 2.4. Substrate Characterization

#### 2.4.1. Nitrogen Adsorption Isotherm 

Brunauer Emmett and Teller (BET) surface area, micro porosity and total porosity were determined from nitrogen adsorption isotherms at 77 K using a Micromeritics ASAP 2020 Physisorption Analyzer (Micromeritics Corp. Norcross, GA, USA). BET theory describes the adsorption of gas particles onto a solid surface based on the assumption of multilayer adsorption, the method often being used by convention to estimate surface area. Samples were measured in triplicate after degassing at 350 °C for 2 h.

#### 2.4.2. Toluene Adsorption Isotherm 

**Figure 1:**
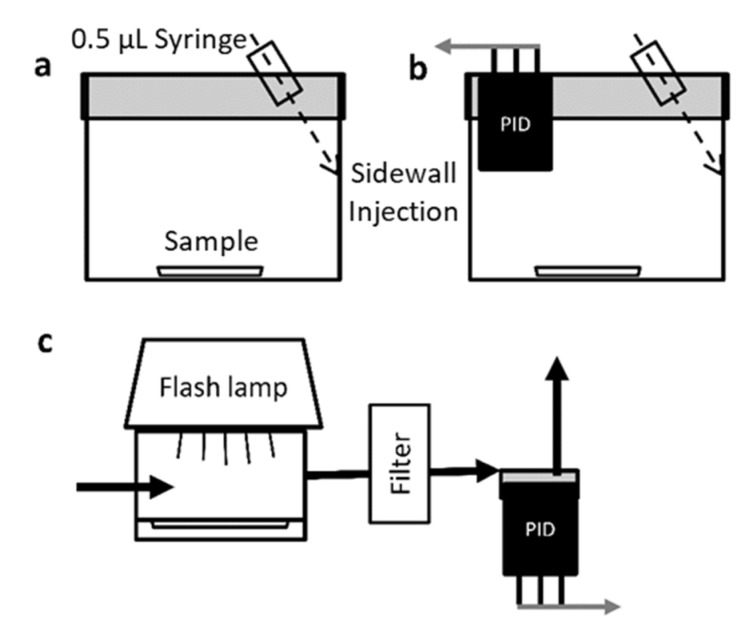
Schematic of test chambers used for sample loading, adsorption isotherm and photothermal desorption experiments. (**a**) 60 mL sample loading chamber with injection port in PTFE-lined lid (**b**) 120 mL adsorption isotherm chamber with photoionization detector (PID) embedded (**c**) photothermal desorption chamber depicted with xenon flash lamp, particulate filter, and PID.

Toluene adsorption isotherms for each sorbent (AC-p, SWNT-p and SWNT-f) were obtained at 23 °C, by placing a known mass of sorbent in the isotherm chamber (Figure 1b). The sorbent was dosed with toluene vapour by injecting a small volume of toluene on the side wall of the isotherm chamber and allowing the system to reach steady state. A more complete description of the dosing technique is given in the sample-loading section. This was repeated until a steady state concentration greater than 150 ppm was achieved.

### 2.5. Sample Loading

To simulate passive adsorption and to evenly dose each sample with a discrete mass of toluene, samples were placed inside a loading chamber (Figure 1a) and indirectly injected with toluene in the same manner as used for toluene adsorption isotherm described above. 435 μg (0.5 μL) of liquid toluene was injected onto the side wall of the jar using a 0.50 microliter syringe. As toluene evaporated it was adsorbed by the sorbent from the vapour phase, ensuring the most homogeneous loading possible which is not achieved with direct liquid dosing. Samples were allowed to stand overnight prior to analysis. This loading technique was validated for all substrates by chemical desorption with CS_2_ using a modified NIOSH 1501 method [5]. The notable modifications of NIOSH 15011 were extraction in a 60 mL glass jar using 5 mL CS_2_ (instead of 1 mL) so that the samples were fully covered with solvent. Analysis was performed by GC-FID using a capillary column (instead of a packed column).

### 2.6. Photothermal Desorption System

#### 2.6.1. Flash Lamp Characterization

A photographic grade xenon flash lamp (Godox 250DI) was mounted above the desorption chamber with the reflector cup directly over the chamber window (Figure 1c). Light energy was measured through the chamber window at the sample tray plane using an Ophir Nova II light power meter and 30A-BB-18 broad band absorption probe (Ophir, North Logan, UT, USA). A plot of light energy vs. capacitor voltage was constructed and 4 capacitor voltages selected that evenly spanned the lamp’s output range. Pulse width at 50% peak height was approximately 4 ms at each capacitor voltage.

#### 2.6.2. Photoionization Detector (PID) Calibration

For these experiments, a field grade photo ionization detector (PID) typical of that used by Industrial Hygienists in direct reading instruments was employed (piD-TECH plus sensor [11], Black, 0–1000 ppm range, Baseline-Mocon Inc., Lyons, CO, USA). This type of PID was selected for its compact size and to demonstrate the feasibility of this desorption technique for in-field measurements with direct reading devices already in use. A two-point daily calibration (0.0 and 100 ppm) was performed using manufacturer guidelines and supplied software. The manufacturer rates sensor accuracy at 3% when calibrated according to their guidelines [47].

#### 2.6.3. System Calibration

After performing the daily calibration, the system as a whole was calibrated at 12 points ranging from 0.019–3.9 µg by injecting toluene vapour into the chamber inlet while under N_2_ flow. This system calibration was performed periodically with calibration checks performed daily. Toluene vapour was injected from either certified calibration gas at 500 ppm toluene in N_2_ (for low mass values) or toluene vapour at the saturation concentration (for high mass values). For the latter, a small amount of liquid toluene was drawn into a gas tight analytical syringe and then the syringe plunger drawn to max with air. The syringe was allowed to sit propped up for 10 min so that liquid toluene remained away from the tip while the headspace saturated with toluene vapour. Desired injection mass was calculated from the Antoine equation and current lab temperature. Prior to injection, a clean needle was installed on the syringe and a small volume of syringe headspace was flushed through the needle to ensure injection of known concentration toluene vapour. No significant difference was found between equal mass injections of headspace saturated vapour and calibration gas (n = 7, *p* = 0.72). The signal to noise ratio at 0.03 ug was approximately 5:1 and this was set as the system limit of detection (LOD).

### 2.7. Desorption

All samples were irradiated once every 60 s for a total of 10 flashes. Each sample was evaluated at each of the 4 selected irradiation energies in triplicate or greater. As a control, preloaded samples were placed in the desorption chamber and loosely covered but not sealed with a reflective aluminium foil cap. Control samples were irradiated as described above at the highest flash energy (4.77 J) such that each control sample was subjected to the same conductive and convective heating mechanisms but not direct radiative heating.

### 2.8. Statistics

ANOVA with Tukey’s HSD post hoc analysis [48] was used to determine differences among substrates and light energies with α = 0.05.

## 3. Results

### 3.1. Substrate Characterization

**Figure 2:**
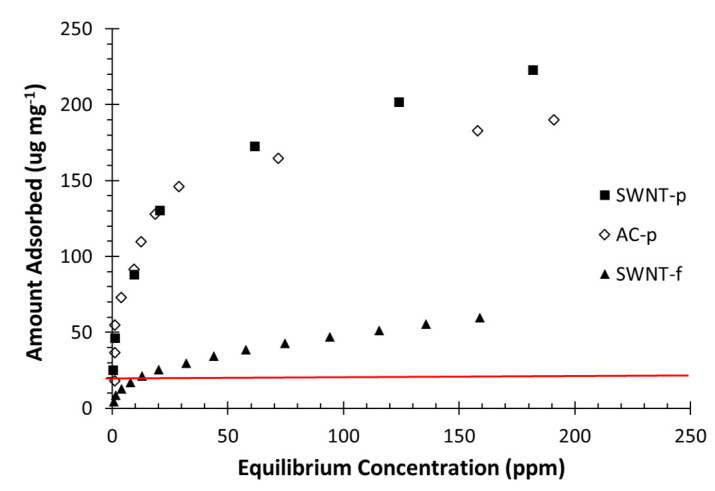
Toluene adsorption isotherms at 23 °C for AC-p, SWNT-p and SWNT-f samples. The horizontal red line represents loading a 20 mg sample with 435 ug toluene.

BET surface area, micropore volume (VμP), total pore volume (VTP), and toluene adsorption capacity are listed in Table 1. Toluene adsorption capacity at 100 ppm equilibrium was determined from toluene adsorption isotherms (Figure 2) as 171, 192 and 48 mg g^−1^ for AC-p, SWNT-p and SWNT-f, respectively. AC-p and SWNT-p are comparable despite the large difference in surface area; however SWNT-f was much lower. It is apparent from these results that processing SWNT powder into felt considerably reduces BET surface area and adsorption capacity. This may be due to dispersing SWNT agglomerates and depositing them as more organized ropes and bundles with less interstitial space. The red horizontal line in Figure 2 represents loading a 20 mg sample with 435 µg of toluene vapour.

### 3.2. Flash Lamp Output

**Figure 3:**
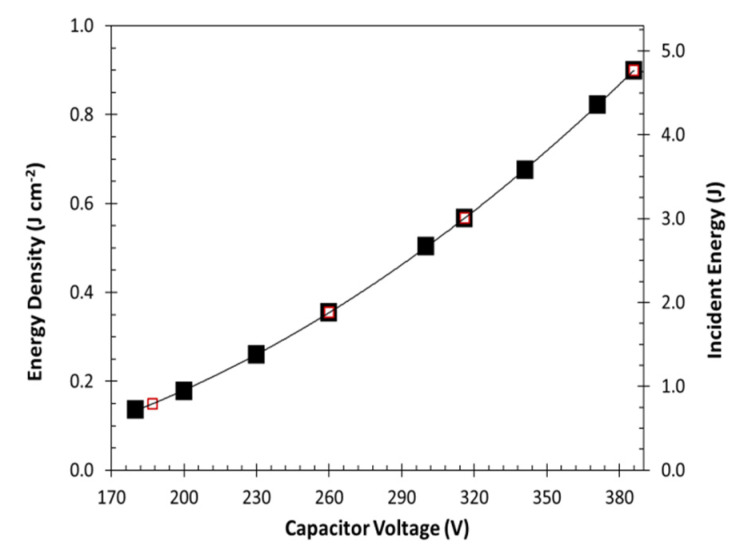
Energy output of photographic xenon flash lamp. Left axis shows energy density (J cm^−2^) at various capacitor voltages. Right axis shows total energy (J) incident to a diameter of 26 mm. Black squares represent lamp settings selected for desorption experiments (0.79, 1.88, 3.01 and 4.77 J).

The relationship between flash lamp capacitor voltage and energy output can be seen in Figure 3. Xenon flash lamps produce broad spectrum UV, visible and infrared light with the UV and IR portions largely absorbed by the lamp’s glass envelope and our borosilicate glass window. Energy density (left axis) as measured by light power meter and incident light energy (right axis) calculated for a disk with 26 mm diameter. The four selected capacitor voltages correspond to incident light energies of 0.79, 1.88, 3.01 and 4.77 J. These are represented by the smaller red outlined, white squares in Figure 3.

### 3.3. Desorption

Mean recovery of toluene using traditional chemical desorption methods was 99.0% ± 1.3, 99.0% ± 1.9 and 94.7% ± 1.7 for AC-p, SWNT-p and SWNT-f, respectively (n = 5). This demonstrates that adsorbed toluene is recoverable from each tested sorbent matrix.

**Figure 4:**
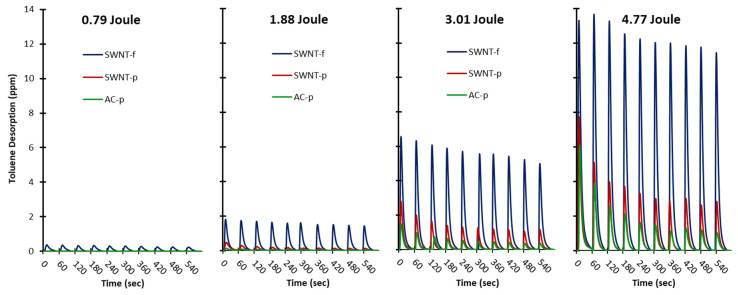
Representative trials with 20 mg sorbent (AC-p, SWNT-p and SWNT-f) pre-loaded with 435 ug toluene. Each sample was irradiated with one flash per minute for a total of 10 flashes, n = 3–4. Irradiation energies were 0.79, 1.88, 3.01 and 4.77 Joules of broad spectrum light from a xenon flash lamp. Axes are held constant to illustrate the difference between flash energies.

Figure 4 shows representative detector responses for each substrate (AC-p, SWNT-p and SWNT-f) irradiated at each energy level (0.79, 1.88, 3.01 and 4.77 J). The Y-axis is held constant to illustrate the difference in desorption at each energy level. Desorption is positively correlated with flash energy. 

**Figure 5:**
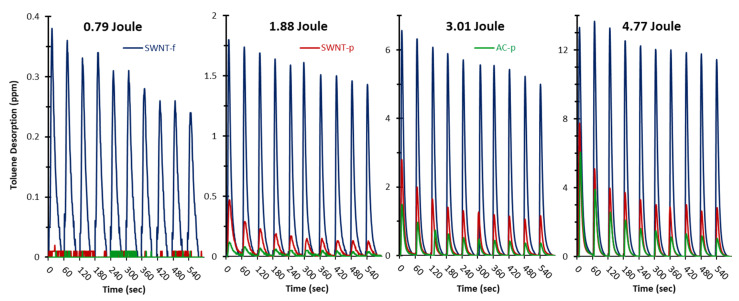
This is the same data as in Figure 4 but axes are scaled for each experiment to illustrate the difference between substrates. There was no observable response in AC-p and SWNT-p at 0.79 J. Representative trials with 20 mg sorbent (AC-p, SWNT-p and SWNT-f) pre-loaded with 435 ug toluene. Each sample was irradiated with one flash per minute for a total of 10 flashes, n = 3–4. Irradiation energies were 0.79, 1.88, 3.01 and 4.77 Joules of broad spectrum light from a xenon flash lamp.

Figure 5 shows the same data as in Figure 4 but with the Y-axis scaled for each trial so that difference between substrates can be seen. In all trials, desorption at a fixed flash energy follows the order SWNT-f > SWNT-p > AC-p. Desorption is more constant in SWNT-f than SWNT-p and AC-p substrates with desorption at the 10th flash relative to the first flash approximately 0.81, 0.23 and 0.18 for SWNT-f, SWNT-p and AC-p, respectively. After eight flashes AC-p and SWNT-p yield levels off but at a much lower fraction than SWNT-f.

**Figure 6:**
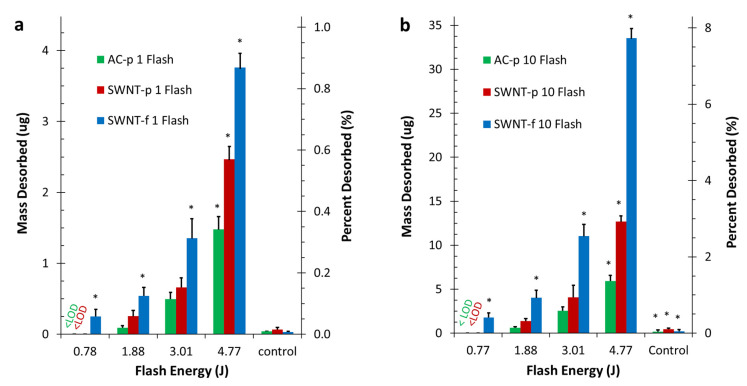
Mean values of desorbed toluene when irradiated at four light energies (0.79, 1.88, 3.01, 4.77 J). Each sample was 20 mg of sorbent preloaded with 435 ug toluene. Left axis is mass of toluene desorbed (ug), right axis is percentage of loaded mass desorbed (%). Controls were flashed with 4.77 J while loosely covered. Asterisk (*) indicate significant differences as described in the text. (**a**) Depicts desorption with a single flash (**b**) depicts cumulative desorption for a 10 flash series.

The average mass desorbed from each substrate by the first flash during PTD trails and the sum of all 10 flashes at 0.79, 1.88, 3.01, 4.77 J and control trials are given in Table 2 and illustrated in Figure 6. At all light energy levels desorption from SWNT-f was significantly greater than from AC-p and SWNT-p. Although desorption from SWNT-p was consistently higher than AC-p in all trials, these differences were only significant 4.77 J. Comparison between control and experiment at 4.77 J found each substrate to be significantly different (*p* < 0.0001). Differences in desorption between light energies were significant at all levels except between the lowest two, 0.79 J and 1.88 J (*p* = 0.711, 0.320, 0.108 for AC-p, SWNT-p and SWNT-f respectively). Figure 6 shows average desorbed mass and desorption percent for the first flash (Figure 6a) and a sum of all 10 flashes (Figure 6b).

## 4. Discussion

### 4.1. Discussion of Desorption Results

The hypotheses of this work were twofold. (1) Irradiation with high intensity light flash can be used to achieve rapid, reliable, partial desorption. (2) SWNT-f will release a significantly larger fraction of analyte than AC-p or SWNT-p when irradiated under similar conditions. The results obtained in this study along with statistical analysis support acceptance of both of these hypotheses. Data shown in Figure 5 and Figure 6 clearly support the first hypothesis regarding rapid, reliable, partial desorption using PTD. PTD is obviously effective, rapid and reproducible.

These same data clearly illustrate greater desorption by SWNT-f under the same irradiation conditions which supports the second hypothesis. Also, these data show SWNT-f has a distinctly different desorption profile than SWNT-p and AC-p during repeated irradiation. Interestingly, SWNT-p and AC-p had similar desorption profiles. Statistical analysis shows that SWNT-f desorption was significantly greater than SWNT-p and AC-p. This is further discussed below as we evaluate two desorption prediction models. In brief, the differences in desorption are best explained by the highly interconnected structure of SWNT-f that better utilizes the excellent thermal conductivity of carbon nanotubes. This is especially clear when contrasting the desorption of SWNT-f with SWNT-p.

**Figure 7:**
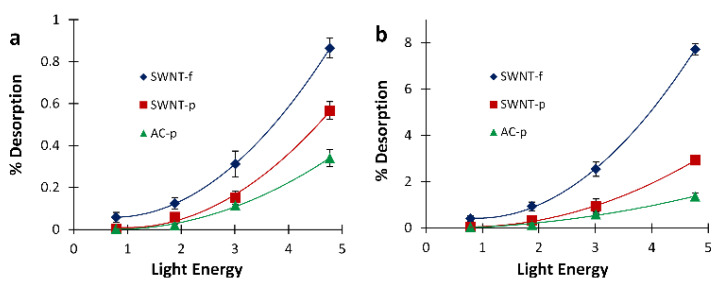
Desorption when irradiated at varied energies. (**a**) First flash desorption at each energy level. (**b**) 10 flash cumulative desorption at each energy level.

Since a future aim of this work is to achieve in-field pre-screening of occupational samples, it is important to quantitatively characterize the relationship between light irradiation with a single flash and analyte desorption. Generally speaking, field devices need to be rugged, reliable, compact and simple; using a single high powered flash to desorb samples for pre-screening assessment will facilitate a simple and compact device. Figure 7 shows desorption results for each substrate fit with a quadratic expression. Using these expressions, the light energy necessary to desorb a given percentage of the sample can be calculated. For example, to desorb 0.5% of the original mass from a SWNT-f substrate, in a single flash, this model predicts a flash energy of 3.73 J is needed. Similarly, the light energy necessary to desorb a larger fraction of the sample in a single flash such as 5% is predicted to be 10.7 J for SWNT-f. Using specific heat capacity estimates for SWNT [42], 10.7 J of input energy would result in a temperature of 446 °C for a 20 mg substrate of SWNT. At this temperature adsorbed compounds may decompose or react with one another which would compromise the original sample. The integrity of the SWNT-f may also be compromised at this desorption temperature, as thermogravimetric analysis of similarly made carbon nanotube sorbents showed 10% mass loss between 295 and 377 °C, and 50% between 451 to 474 °C, dependent upon the carbon nanotube species used for sorbent fabrication [9]. Therefore, an in-field pre-screening tool would need to use a single flash that does not compromise the original sample nor the sorbent matrix. More simply put, the flash energy needs to be lower so that the sorbent temperature does not overheat.

If a higher desorption fraction is needed, a better approach would be applying multiple flashes at lower energies that do not compromise analyte integrity. This could be accomplished in a field instrument, but we foresee this more likely to be used with in-lab analysis coupled with a GC or similar analytical instrument. Using the 10 flash summed data in Figure 7b for SWNT-f, the flash energy needed to desorb 1% and 5% of the sample is calculated to be 2.0 and 4.0 J, respectively. These input energies should cause a temperature increase of 112 and 200 °C, respectively, which poses minimal threat to analyte and sorbent integrity.

### 4.2. Application to Occupational Exposure Assessment

With this system, we desorbed 3.76 ug out of 435 ug (0.86%) from SWNT-f substrates with a single flash at 4.77 J. The limit of detection of our system was established as 0.03 ug (5:1 signal to noise ratio) during calibration. From the LOD of our system, a reliable quantitation limit (RQL) of 0.09 ug was established (3X LOD). Therefore this system is expected to be capable of quantifying an unknown sample with as little as 10.5 ug toluene. This is approximately the mass collected in 15 min at 6 ppm by a 3M passive monitor. Several system changes could be implemented to improve sensitivity such as: reducing desorption chamber dead volume and reducing chamber flow rate. These changes would decrease residence time within the chamber and minimize readsorption of analytes by the sorbent. These changes would also reduce the dilution of desorbed analytes and result in a more intense sensor response. Another simple system improvement is using a PID with lower sensing range. The PID used in this study was a high range PID rated up to 1000 ppm, a lower range PID would improve sensitivity and lower the LOD and, therefore, RQL. With these simple improvements, an in-field pre-screening unit capable of quantifying a 15 min, 1 ppm exposure is easily achievable. Since the remaining >99% of the sample is still on the original sorbent, the sample could still be sent to a laboratory for conventional analysis with negligible sensitivity loss.

### 4.3. Developing a Desorption Prediction Model

Since each flash depletes the mass available to desorb by subsequent flashes, we should not expect the mass yield to remain constant with continued flashing. Instead, we should expect the desorption ratio to remain constant given the following three assumptions. (1) Analyte concentration within the sorbent is homogenous, (2) Sorbent heats uniformly, and (3) Analyte is not destroyed by irradiation (i.e., no pyrolysis or photolysis). From this, a simple expression can be used to predict cumulative desorbed mass when a given number of flashes are applied and the first flash desorption ratio is known.

(1) $$M_{D}=M_{o}-M_{o}{\left(1-r\right)}^{n}$$

where: M_D_ = cumulative desorbed mass, Mo = initial analyte mass in sorbent, r = desorption ratio of first flash (M_D_/Mo), and n = flash number.

**Figure 8:**
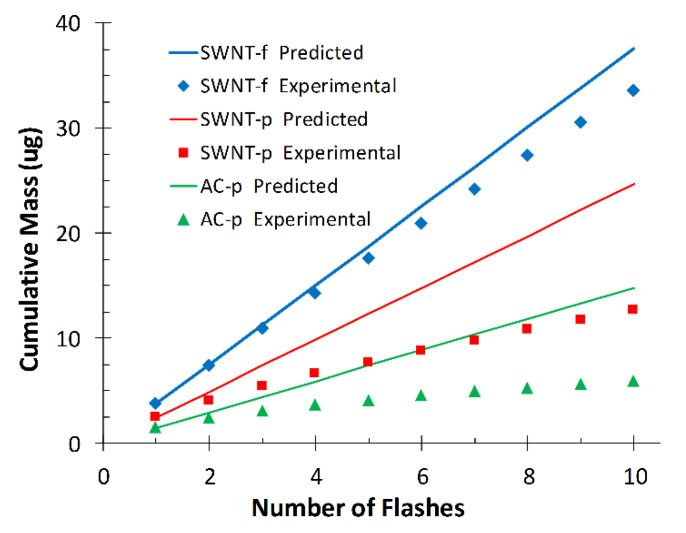
Cumulative desorbed mass predicted by model (solid lines) and experimental data.

Figure 8 shows the experimentally observed cumulative desorption of each substrate compared to that predicted by the model (Equation (1)).

For SWNT-f the model is in close agreement with experimental data after 10 flashes (112%), however not so for AC-p (249%) and SWNT-p (194%). This is most likely due to violation of our second assumption; that the sorbent heats uniformly. Powdered samples are unlikely to have efficient thermal conduction through their many layers; as such, the same energy load is being shared with less carbon mass in the upper layers. The temperature is higher at the surface and lower in the bulk than estimated from specific heat calculations. The analyte is quickly depleted from a thin layer on the top of the substrate which is evident from the rapidly decreasing yield with subsequent flashes (Figure 4 flashes 1–6). This depleted layer is minimally resupplied with toluene by diffusion of toluene from deeper within the sorbent. In the 60 s between flashes a small amount of toluene replenishes the available pores in the surface layers which accounts for the levelling-off in response after seven to eight flashes. Conversely, the more dense and interconnected structure of SWNT-f utilizes the excellent thermal conductivity of carbon nanotubes so that energy absorbed at the surface is more efficiently conducted into the bulk and transferred to sorbent that is not directly irradiated. The entire substrate heats uniformly and depletes analyte in a proportional manner. The small deviation from the model by SWNT-f (12% over 10 flashes) may be due to the silver membrane acting as a heat sink on the bottom of the substrate and creating a thermal gradient within the substrate thus causing a mild violation of our second assumption, uniform heating. This mild violation could also be the result of a thermal gradient created by horizontal diffusion of heat across the SWNT-f surface, resulting in a reduction of thermal energy penetrating vertically through the material. A similar phenomenon was observed by Shedd et al., in their related study on the thermal characteristics of self-supporting SWNT sorbents used with photothermal desorption [44].

Using experimental data for each substrate at 4.77 J, cumulative desorption (M_D_) at flash number can be described by a linear function and used to calculate an empirically observed desorption ratio (r) which tremendously improves the model for AC-p and SWNT-p, but only moderately improves SWNT-f.

(2) $$M_{D}=\frac{an+b}{M_{o}}$$

where: a = slope of cumulative mass vs. flash number plot, and b = mass intercept of cumulative mass vs. flash number plot.

By rearranging Equation (1) to solve for M_o_ we can use the modified model to predict initial mass (M_o_) at varied number of flashes (1–10).

(3) $$M_{O}=\frac{M_{D}}{1-{\left(1-r\right)}^{n}}$$

where: r is calculated using Equation (2) and is confined to a specific flash energy.

**Figure 9:**
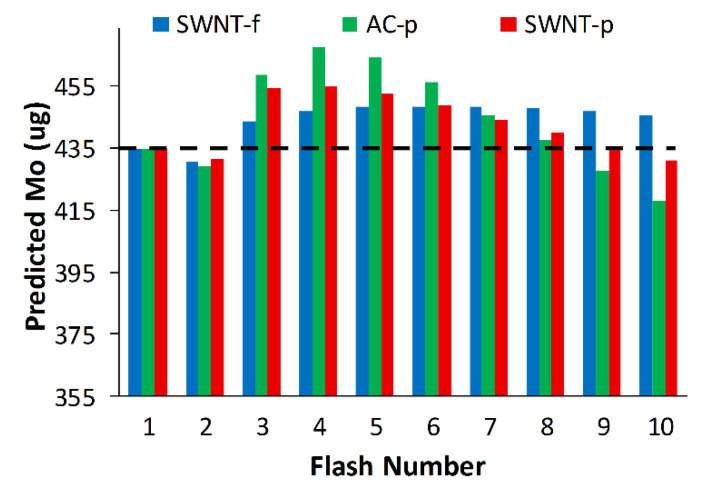
Modified model predictions of initial mass (Mo) using cumulative desorbed mass and flash number. True initial mass = 435 ug toluene which is represented by the dashed black line.

Using the modified model to predict the initial mass of each sorbent, we find that predictions of M_o_ are within 7.5%, 4.6% and 3.1% of the true initial mass (435 µg toluene) for AC-p, SWNT-p and SWNT-f, respectively (Figure 9). Although the large deviation between initial model predictions and experimental results for AC-p and SWNT-p are correctable with this modified model, small variations in material properties such as grain size or packing density will greatly affect thermal conductivity which could drastically alter desorption and thus negate the accuracy of the correction. For this reason and the impracticality of handling fine carbonaceous powders, AC-p and SWNT-p substrates are not recommended for use as passive sampler media nor for photothermal desorption. Conversely, SWNT-f has more uniform and higher desorption yields, good agreement with our simple model, better agreement with the modified model, and is much safer to handle. Therefore, we recommend SWNT-f substrate for further evaluation with photothermal desorption.

### 4.4. Limitations

Only one VOC (toluene) was evaluated in this study. Toluene was selected as a representative VOC and since this is a thermal process, what works for one compound should work for others; however, we expect desorption ratios of other VOCs to be unique based on vapour pressures, heat of vaporization, adsorption enthalpies, molecular mass and molecular size. Trials with other chemical classes should be performed individually and ultimately as a mixture of compounds.

From the assumptions of our model, desorption fraction will remain constant with loading factor. This should be verified experimentally.

For the purpose of achieving baseline resolution from each flash, samples were irradiated once every minute for 10 min. In future applications, use of rapid irradiation would be desirable, but it is uncertain whether rapid irradiation will produce desorption equivalent to flashes administered once per minute. This is especially relevant for AC-p and SWNT-p substrates which appear to approach a diffusion limit after seven to eight flashes. There is no indication of this limitation in SWNT-f which is another advantage of this substrate over AC-p and SWNT-p.

In an effort to use common, low-cost components that could be adapted for an in-field pre-screening device, we selected a photographic grade flash lamp. Flash energy was the independent variable in these experiments, but another variable of interest could be pulse rise time. This was measured and found to be consistent (4 ms) at each flash energy in these experiments, but was not tunable with this system. Also, we were unable to measure or obtain a representative spectral distribution from the manufacturer. Since this is a typical photographic grade xenon flash lamp its spectral distribution was assumed to be similar to other xenon flash lamps [49].

The material cost of SWNTs is substantially higher than that of activated carbons used for air sampling and higher than the sorbents used for thermal desorption. In the past decade the industrial use of carbon nanotubes has increased which has increased the supply and is decreasing the costs of analytical quality SWNTs to some extent. As production techniques become more scalable and demand increases we expect these material costs to continue to fall. Regardless, the SWNT felts are expected to be reusable for many cycles and the quantity of material used is quite small (20 mg in this study). Future studies should explore the suitability of lower cost, perhaps lower quality CNTs for PTD applications.

The present study used indirect vapor dosing to determine the toluene adsorption of our sorbents. Although dosing chambers were filled with ambient air, the exposure to humidity in the closed system was potentially less than that expected from the open environment of personal sampling. In future, in field studies, the adsorption capacity may be reduced as excess water occupies adsorption sites within the sorbent. That said, we do not expect adsorbed water to have an observable effect on analyte mass recovery by photo flash.

## 5. Conclusions

In this work a common photographic grade xenon flash lamp was demonstrated to desorb toluene from three substrates; AC-p, SWNT-p and SWNT-f. Processing SWNT-p into SWNT-f reduced the BET surface area, microporosity and toluene adsorption capacity, but increased the overall porosity. It was also observed that SWNT-f was most amenable to photothermal desorption of the three substrates studied. Significantly larger fractions of toluene were released from SWNT-f than from AC-p and SWNT-p under the same conditions and the release rate was much more consistent within SWNT-f upon repeated flashes. Photothermal desorption showed a wide desorption range; from a single flash the range was 0.001–0.86% depending on substrate type and flash energy, from a 10 flash series as much as 7.7% was desorbed. A positive correlation exists between flash energy and toluene desorption which was modelled. The presented modified model predicted initial toluene mass within 7.5%, 4.6% and 3.1% of the pre-loaded mass (435 µg) for up to 10 flashes for AC-p, SWNT-p and SWNT-f, respectively. Lastly, the photothermal desorption system used in this study showed sufficient sensitivity to quantify as low as 10.5 µg of toluene using SWNT-f substrates. Reducing system flow, using a more sensitive PID, and decreasing the chamber dead volume could easily lower the system sensitivity by 10-fold or more. Photothermal desorption has tremendous potential for laboratory use as a fast, enhanced-sensitivity injection technique for GC as well as in-field pre-screening of VOC exposure samples.

If used as an injection technique for GC, photothermal desorption promises a dynamic analytical range far surpassing that of liquid injection or thermal desorption and sensitivity approaching that of thermal desorption. If used to pre-screen VOC exposure samples, this technique could allow more expansive worker sampling since those samples with very low exposures could be discarded and only samples indicating potential over-exposure could be sent to an accredited laboratory for full analysis. Further investigation is needed to explore application of this technique such as investigating other VOCs and lower analyte loading.

## 6. Patents

Provisional patent 63/251,286 was filed on 10/01/2021 to protect the intellectual property herein.

## Figures and Tables

**Table 1:** Surface properties of activated carbon powder (AC-p), single-walled carbon nanotube powder (SWNT-p) and felt (SWNT-f) substrates using nitrogen adsorption at 77 K.

	BET [m^2^g^−1^]	VµP [cm^3^g^−1^]	VTP [cm^3^g^−1^]	Toluene Adsorption Capacity [mg g^−1^]
AC-p SWNT-p SWNT-f	815 980 345	0.20 0.39 0.04	0.68 0.59 0.88	171 192 48

**Table 2:** Average analyte desorption from substrates when irradiated at varied light energies. Limit of detection (LOD) = 0.03 ug.

	Mass Desorbed (ug) when Irradiated at:				
	0.79 J	1.88 J	3.01 J	4.77 J	Control
AC-p 1st Flash	<LOD	0.092	0.498	1.480	0.039
SWNT-p 1st Flash	<LOD	0.255	0.662	2.466	0.068
SWNT-f 1st Flash	0.251	0.542	1.356	3.760	0.030
AC-p 10 Flashes	<LOD	0.609	2.563	5.939	0.165
SWNT-p 10 Flashes	<LOD	1.370	4.092	12.70	0.448
SWNT-f 10 Flashes	1.793	4.020	11.04	33.53	0.190

## Data Availability

The authors confirm that the data supporting the findings of this study are available within the article. Additional question concerning the data presented may be addressed to the corresponding author [Claudiu T. Lungu].
